# Fecaloma presenting as huge abdominal mass

**DOI:** 10.1002/jgh3.12221

**Published:** 2019-06-25

**Authors:** Mushtaq A Khan, Hilal A Dar, Altaf H Shah, Gul Javid, Bhagat Singh, Nadeem A Sheikh, Aadil Ashraf

**Affiliations:** ^1^ Department of Gastroenterology Sher‐i‐Kashmir Institute of Medical Sciences Kashmir India

**Keywords:** clinical gastroenterology in the elderly, clinical intestinal disorders, colonic motility and disorders, intestinal ischemia

## Abstract

Fecal impaction is common in elderly, bed ridden, schizoaffective patients on antipsychotics. Intestinal obstruction due to distal colonic fecaliths is rare as it is amenable to digital manual evacuation and enemas. Our patient presented with abdominal distention, with last bowel evacuation reported 3 months ago. Computed tomography (CT) abdomen demonstrated a huge sigmoid fecalith causing bilateral hydronephrosis. He was managed through laparotomy with sigmoid colon resection and end colostomy.

## Graphical abstract

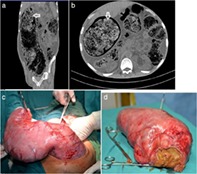

We present the case of a fecaloma presenting as a huge abdominal mass.

## Introduction

Fecal impaction is common in bedridden and debilitated elderly patients. Intestinal obstruction due to prolonged fecal impaction in the distal colon is rare as it is amenable to manual evacuation and enemas.^1^ Patients with schizoaffective disorders and those on antipsychotic medication have a high risk of fecal impaction. Neglected fecaloma can lead to intestinal obstruction, compartment syndrome, stercoral colitis, and colonic perforation. Surgical management for such cases is recommended as colonic perforation has been reported with high mortality rates of 30–60%.^2^, ^3^ We present a case of large bowel obstruction due to large fecalith manifesting as a huge abdominal mass.

## Case report

A 48‐year‐male, schizophrenic on clozapine, presented with complaints of progressive abdominal distention, mild abdominal discomfort, and recurrent episodes of vomiting for 10 days. He had not passed stool for the last 3 months. Previously, the patient used to move bowel after a week and occasionally used enemas for constipation. During these 3 months, the patient took laxatives and enemas but did not pass stool. Clinical examination demonstrated a pulse of 89 beats/min, blood pressure (BP) 110/80 mmHg, and respiratory rate of 14 cycles/min. Examination showed a hugely distended abdomen with a firm irregular mass occupying the left iliac, hypogastrium, right Iliac fossa, and right lumbar quadrant of the abdomen. Bowel sounds were absent, and no bruit was heard. On rectal examination, anal tone was normal, and a hard mass was felt in the rectum.

**Figure 1:**
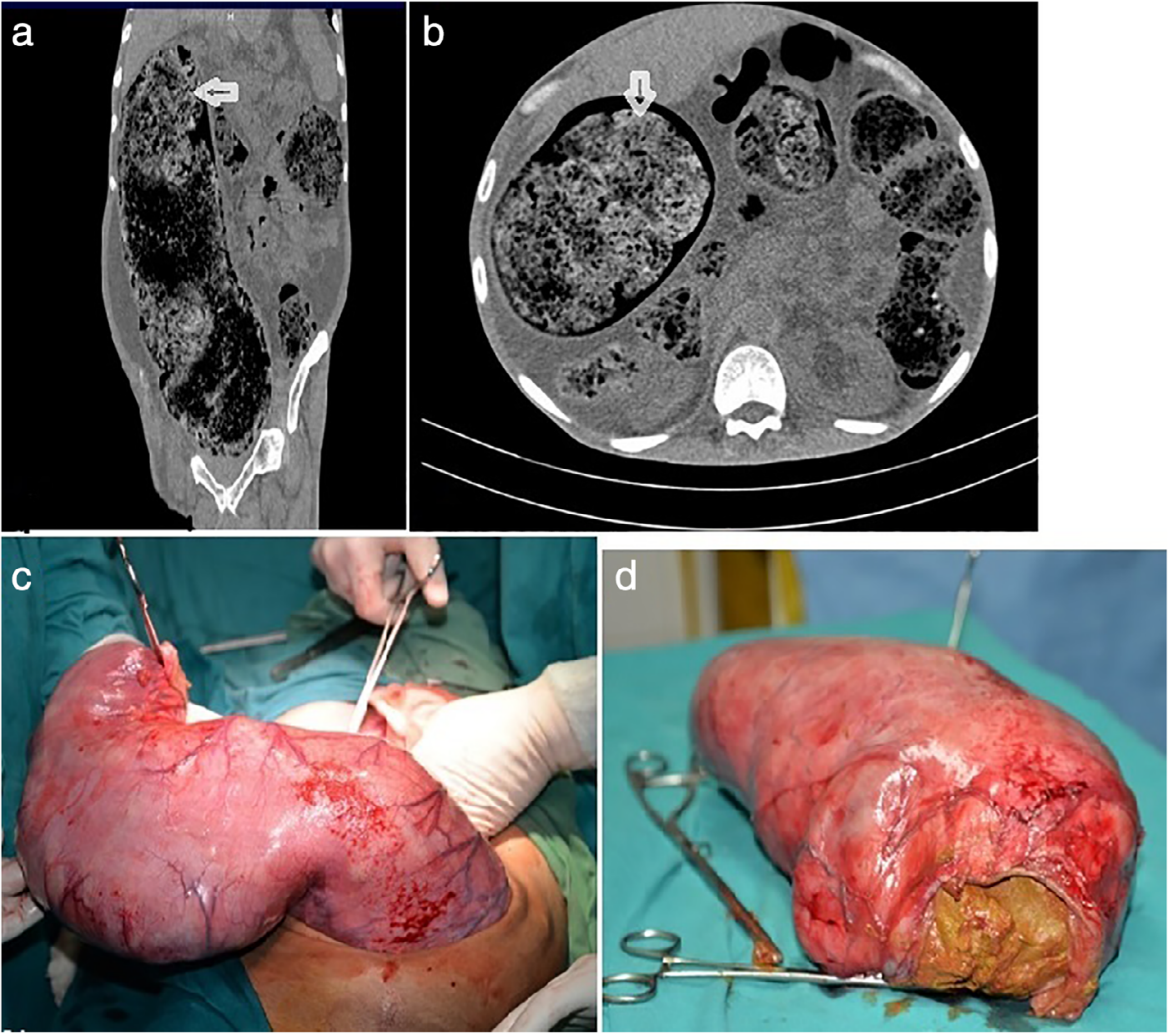
(a) Computed tomography (CT) abdomen showing dilated sigmoid colon, filled with a hyperdense mass with air foci, shown by arrow; (b) CT abdomen (transverse section) demonstrated dilated large bowel, filled with hyperdense material; (c) intraoperative findings showed markedly dilated left‐sided colon, with approximate diameter of 30 cm; and (d) resected sigmoid colon filled with fecal matter and fecaliths.

Complete blood count was within normal limits. Serum chemistry demonstrated urea, 80 mg/dL; creatinine, 2.37 mg/dL; and serum albumin, 2.82 gm/dL. Abdomen radiograph showed a huge mass occupying almost the whole abdomen with no gas in the bowel loops. Computed tomography (CT) abdomen showed grossly distended rectosigmoid colon filled with hyperdense material suggestive of fecal matter and dilated descending colon (Fig. 1a,b).

In view of the marked distension of the sigmoid colon with impending perforation, the patient was subjected to laparotomy. Intraoperative findings indicated a markedly distended sigmoid colon approximately 30 cm in diameter containing hard fecal matter (Fig. 1c,d). The sigmoid colon was resected, and a temporary colostomy was performed.

## Discussion

Fecal impaction is defined as hard stool in the intestinal tract not evacuated spontaneously.^4^ It may lead to intestinal obstruction and should be perceived as an emergency. It can lead to stercoral colitis, and so far, fewer than 150 cases have been reported in the literature. Fecal impaction leading to stercoral colitis was first described by Berry in 1894.^5^ Stercoral colitis is a rare inflammatory process involving the colonic wall secondary to fecal impaction and may lead to colonic ischemia, stercoral ulcers, and perforation. Fecaloma most commonly develops in the distal colon as stool get harder due to poor hydration. It can lead to pressure necrosis of the bowel wall by impairing the blood circulation. This results in colonic wall inflammation and ulcers, leading to perforation and fecal peritonitis in some cases. The most common sites of perforation due to fecaloma causing stercoral colitis are the antimesenteric border of the rectosigmoid region and anterior rectal wall.^6^, ^7^ Elderly, debilitated, and bedridden patients are more prone to fecal impaction, but cases have been reported in those belonging to the 15–65 years age group. Patients with schizoaffective disorders on antipsychotic, anticholinergic, tricyclic antidepressants, and antiserotonergic medication are at higher risk. Fecal impaction in the distal colon can be managed by manual evacuation, bowel washes, and enemas. Prolonged fecal impaction leads to a distended colonic wall with stercoral ulcers, inflammation, and have good chances of perforation with manual evacuation and enemas. For these patients, surgical management is recommended. Laparotomy with resection of diseased colon and end colostomy with Hartman's procedure are recommended. It is important for treating doctors to have high clinical suspicion for at‐risk patients with fecal impaction. Prolonged fecal impaction may lead to colonic perforation with a mortality rate of 30–60%, which can be prevented by early and adequate treatment of constipation and fecal impaction in high‐risk groups, especially in elderly patients with multiple comorbidities.
